# Divergent microbial communities in groundwater and overlying soils exhibit functional redundancy for plant-polysaccharide degradation

**DOI:** 10.1371/journal.pone.0212937

**Published:** 2019-03-13

**Authors:** Martin Taubert, Jan Stähly, Steffen Kolb, Kirsten Küsel

**Affiliations:** 1 Aquatic Geomicrobiology, Institute of Biodiversity, Friedrich Schiller University Jena, Jena, Germany; 2 Microbial Biogeochemistry, RA Landscape Processes, Leibniz Centre for Agricultural Landscape Research (ZALF), Müncheberg, Germany; 3 German Centre for Integrative Biodiversity Research (iDiv) Halle-Jena-Leipzig, Leipzig, Germany; CSIR, INDIA

## Abstract

Light driven primary production by plants is the main source of biomass in terrestrial ecosystems. But also in subsurface habitats like aquifers, life is fueled largely by this plant-derived biomass. Here, we investigate the degradation of plant-derived polysaccharides in a groundwater microbiome to identify the microbial key players involved, and compare them to those from soil of the groundwater recharge area. We quantified the activities of enzymes degrading the abundant plant polymers starch, cellulose and hemicellulose in oligotrophic groundwater samples, despite the low cell numbers present. Normalized to 16S rRNA gene copy numbers, these activities were only one order of magnitude lower than in soil. Stimulation of the groundwater microbiome with either starch or cellulose and hemicellulose led to changes of the enzymatic activity ratios, indicating autochthonous production of enzymes in response to the plant polymers. Furthermore, DNA stable isotope probing with ^13^C labelled plant polymers allowed us to identify microbes involved in the degradation of these compounds. In (hemi)cellulose microcosms, *Bacteroidia* and *Candidatus* Parcubacteria were active, while the active community in starch microcosms mostly comprised *Candidatus* Saccharibacteria, *Cytophagia*, and *Actinobacteria*. Not a single one of the active OTUs was also found to be labelled in soil microcosms. This indicates that the degradation of plant-derived polysaccharides in groundwater is driven by organisms completely distinct from those active in soil. The involvement of members of the candidate phyla *Cand*. Parcubacteria and *Cand*. Saccharibacteria, organisms known to be abundant in groundwater, in plant-derived organic matter degradation might strongly impact subsurface carbon cycling.

## Introduction

In the subsurface, the light-driven primary production dominating surface environments is missing, leading to oligotrophic conditions. Microbial life in pristine groundwater ecosystems hence is dependent on lithoautotrophy [1], ancient deposited organic carbon released from rocks [2], and on surface-derived allochthonous carbon inputs [3–5]. The major part of organic carbon produced on the surface is derived from plants. An estimated 98 Gt of terrestrial plant derived organic carbon is annually released back into the atmosphere as CO_2_ [6], but information about the contribution of subsurface environments to this process is lacking. Biopolymers such as cellulose, hemicellulose, and starch act as structural components or storage compounds in plants. These plant polymers make up 35 to 50% of the plant dry mass, and thus form a significant reservoir of organic carbon [7]. The vertical fluid flow connecting surface habitats to the aquifers allows the transport of dissolved as well as particulate plant derived organic carbon into the groundwater [8–11]. Hence, insoluble plant polymers as well as partially degraded and soluble polymeric material can represent relevant carbon sources for heterotrophic microorganisms, thereby shaping the aquifer microbiomes [12–14]. The relative importance of surface inputs compared to lithoautotrophy and rock-derived organic carbon, and the impact of subsurface heterotrophy on the global carbon cycle, however, are largely unknown.

The microbial utilization of plant polymeric carbohydrates requires an extracellular degradation step to allow the uptake of the resulting mono- and disaccharides into the microbial cells [15]. This extracellular degradation is achieved by the release of hydrolytic and other polysaccharide-degrading enzymes. Such exoenzymes often work together and form ternary complexes with their polymeric substrates [7, 15]. In plant-derived polymeric carbohydrates, different bonds between monomers occur, such as α-1,4-glycosidic and α-1,6-glycosidic bonds in starch, or β-1,4-glycosidic and various other bonds in cellulose and hemicellulose. The hydrolysis of these different kinds of bonds requires different enzymes [7, 15]. In the initial step of degradation, endo-acting enzymes cleave random internal bonds of the polymer chain, producing more termini, while exo-acting enzymes cleave di- or oligomers from the termini. Resulting oligo- and dimers are further hydrolyzed by enzymes often attached to the outside of the bacterial cell wall: maltose from starch is cleaved by α-glucosidases, cellobiose from cellulose by β-glucosidases, and xylobiose and other β-D-xylans from hemicellulose by β-xylosidases [7, 15, 16]. The formed monomers and dimers are then taken up by the microorganisms and serve as carbon and energy source. As a result of the extracellular degradation process, the formed oligosaccharides are also available to other microorganisms than those producing exoenzymes [17].

In this study, we explored the microbial metabolic potential for plant polysaccharide degradation in an oxic limestone aquifer of the Hainich Critical Zone Exploratory (CZE), Germany, where the flow of pristine groundwater can be followed from its origin through the subsurface downhill [8]. Previous studies have shown differences in microbial community composition between soil and groundwater, specifically revealing a high relative abundance of organisms from the candidate phyla radiation in groundwater ecosystems [18]. Belonging to the uncultured majority of microorganisms [19, 20], the functions of these *Bacteria* have remained largely uncharacterized. However, it is also known that soil organisms can be transported into groundwater [10, 21]. Our goal was to explore whether the microbial key players for plant polymer degradation differ between groundwater and soil of the Hainich CZE, and how putative differences affect this ecological function in the two habitats. First, to characterize the functional patterns of plant polymer degradation, we measured enzyme activities in groundwater samples with a fluorescence assay using methylumbelliferon-(MUF)-conjugated substrates, and compared them with soils from the groundwater recharge area. Second, we performed a DNA stable isotope probing (DNA-SIP) experiment, employing ^13^C labelled starch, cellulose, and hemicellulose, to identify the groundwater microorganisms using these polysaccharides as carbon sources. In DNA-SIP, a separation of ^13^C labelled (heavy) and unlabelled (light) DNA is possible by density gradient ultracentrifugation [22]. Traditionally, in DNA-SIP experiments the presence or absence of OTUs in the ^13^C heavy fraction and the ^12^C heavy fraction is evaluated to identify labelled microbial taxa [22], e.g. microbial taxa are deemed to be labelled when their DNA is present in the heavy fraction of ^13^C samples but absent from the heavy fraction of ^12^C samples. Increasing complexity of the microbial community investigated, however, leads to a low specificity of this ‘heavy SIP’ method [23]. Recent studies started to use statistical approaches and qPCR data to validate enrichment of OTUs in the ^13^C heavy fraction [24, 25]. These approaches rely on amplicon sequencing of all density gradient fractions, which amounts to hundreds of samples in a single SIP experiment. Even with the steady decline in sequencing costs, such approaches are often financially not feasible. Here, we restricted our analysis to the light and heavy fraction of ^12^C and ^13^C samples, but applied a novel statistical approach to identify labelled microbial OTUs. Furthermore, we hypothesized that incubation with the different polymeric carbohydrates would result in production of enzymes by the groundwater microbiome, leading to changes in enzyme activity patterns, and thus, we also determined activities of polysaccharide cleaving enzymes at the end of the SIP incubations. Parallel SIP incubations with soil samples eventually allowed us to compare the active plant polymer utilizing microorganisms between surface and subsurface.

## Results and discussion

### Groundwater and soil sampling

We collected groundwater and soil from a common hydrogeological setting by using the infrastructure provided by the Hainich CZE [8, 26]. Groundwater was obtained from well H41 accessing an oxic aquifer assemblage, and is characterized by average chemical values of 5.0 ± 1.5 mg L^-1^ dissolved oxygen (DO), pH 7.2, < 0.1 mg L^-1^ ammonium, 1.9 ± 1.5 mg L^-1^ dissolved organic carbon (DOC), and 70.8 ± 12.7 mg L^-1^ total inorganic carbon (TIC) [26–28]. Due to the low cell numbers (10^5^ cells mL^-1^) in the highly oligotrophic groundwater [29], a high volume filtration of 5,000 L through a 0.3 μm glass fiber filter was carried out as previously described [28, 30] to enrich microbial biomass. For comparison, soil samples from a European beech (*Fagus sylvatica* L.) forest resembling the groundwater infiltration area [26] were investigated. The soil was previously classified as Rendzic Leptosol-Cambisol [26], with a pH of 3.8 to 4.0, organic carbon content of 14 to 26 g kg^-1^, and C:N ratio of 11 to 12 [31].

### Polysaccharide cleaving enzyme activities in groundwater

**Fig 1:**
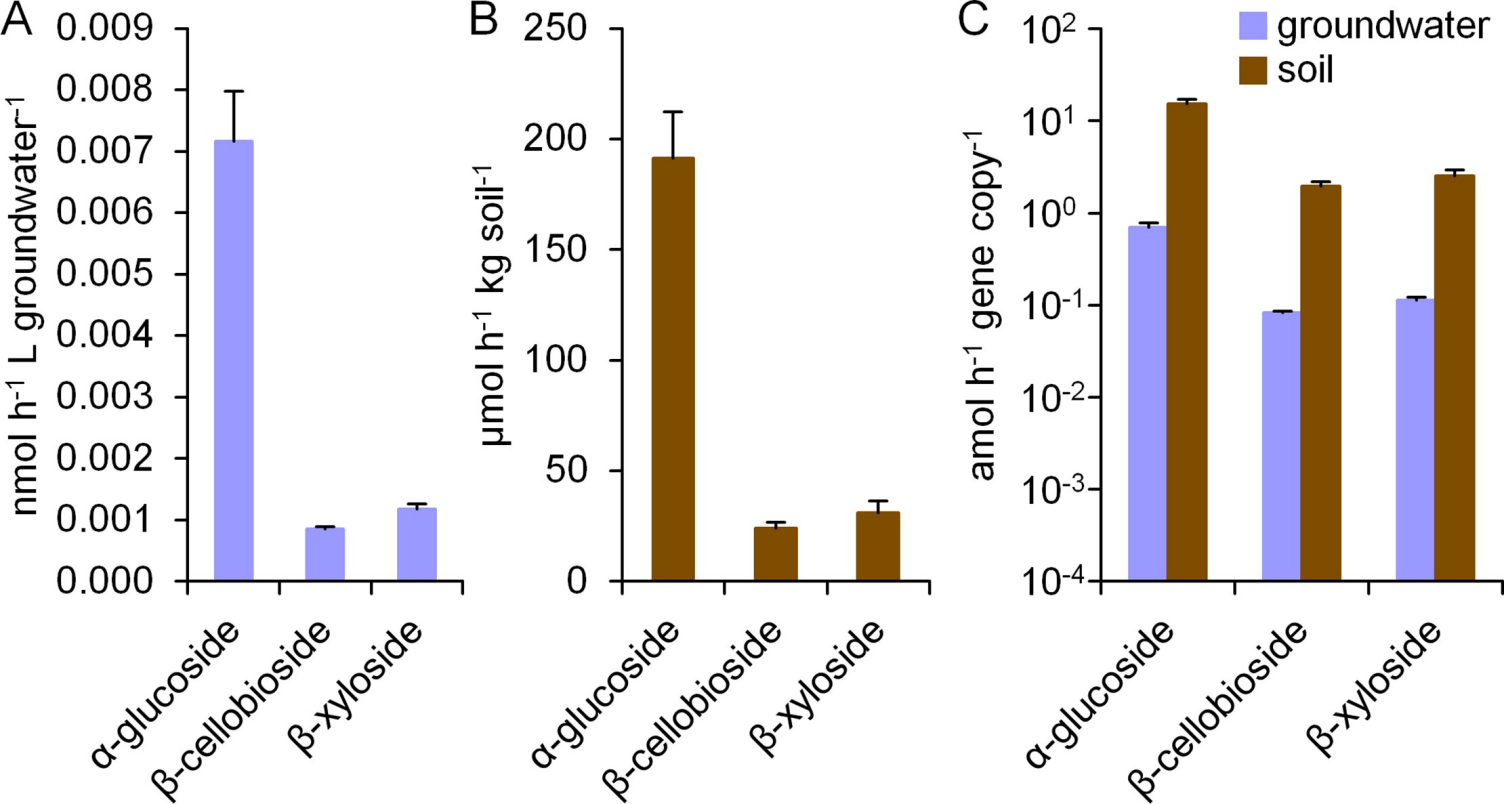
Enzyme activities in groundwater and soil samples. Activities on α-glucoside, β-cellobioside and β-xyloside measured in a fluorometric assay using MUF substrate analogues for (A) groundwater samples from well H41 of the Hainich CZE, and (B) soil samples from the nearby beech forest. (C) For comparability, enzyme activities were normalized to bacterial 16S ribosomal gene copy numbers determined by qPCR. Values represent averages of enzyme activities in groundwater samples (blue bars, 3 technical replicates) and in soil samples (brown bars, 3 biological replicates), and error bars depict standard deviations.

To characterize the functioning of the groundwater microbial community in plant polymer degradation, we used the MUF-conjugated substrates α-glucoside to assess the degradation potential for starch, as well as β-cellobioside and β-xyloside to assess the degradation potential for cellulose and hemicellulose. Enzyme activities in groundwater samples obtained from the Hainich CZE well H41 were assayed using a fluorometric method [32, 33]. This highly sensitive approach enabled us to detect the extremely low enzyme activities with all three substrates in the groundwater, even though these were several orders of magnitude lower than those measured in soil samples (Fig 1A and 1B). A normalization to copy number of bacterial 16S RNA gene determined by quantitative polymerase chain reaction (qPCR) (S1 Table) surprisingly revealed similar levels of enzyme activity per gene copy between soil and groundwater (Fig 1C), with no significant difference between the activity ranges observed (p = 0.287, Student’s *t*-test). The enzyme activities in groundwater were in the range of 0.07 to 0.8 attomol (amol; 10^−18^ mol) h^-1^ gene copy^-1^ compared to 2 to 15 amol h^-1^ gene copy^-1^ for soil. Reported soil activities for the three substrates investigated, α-glucoside, β-cellobioside, and β-xyloside, typically range from the nmol to lower μmol range per g soil and hour [34–37]. The activities observed in the beech forest soil samples described here were near the lower end of this range. In contrast, only little data on polysaccharide degrading enzyme activities in groundwater exist. A response of the synthesis of extracellular enzymes to changes of nutrient availability was previously demonstrated by Kolehmainen and colleagues in artificial groundwater recharge systems [38]. The reported activities in natural groundwater on α- and β-glucoside were between 0.02 and 0.05 amol h^-1^ cell^-1^. Assuming that each bacterial cell contains two to five copies of the 16S rRNA gene [39], these data are around one order of magnitude lower than the 0.07 to 0.8 amol h^-1^ gene copy^-1^ found in our study.

Interestingly, the ratios between activities on the three substrates measured were almost identical for groundwater and for soil, revealing a similar functionality for plant polymer degradation. The activities on β-cellobioside and β-xyloside were approximately in the same range, but the activity on α-glucoside was one order of magnitude higher in both habitats. β-glucosidases are known to exhibit relatively low *k*_cat_ values [40], potentially explaining the lower activities on β-cellobioside. Furthermore, starch is relatively accessible to enzymatic attack compared to cellulose and hemicellulose [15], and hence microorganisms might preferentially produce enzymes for starch degradation when different plant polymers are present. Previous studies on different soil types reported either activities in the same range for β-cellobioside and β-xyloside [37, 41], or a two- to three-fold activity for β-cellobioside compared to β-xyloside [36]. The activity on α-glucoside was reported to be twice as high as that of β-xyloside in a study on tropical soils [34]. In the hyporheic zone of the Queets River (WA, USA), up to 6 times higher activity on α-glucoside than on β-xyloside have been observed [42], fitting with the higher observed activity on α-glucoside in our study. However, the comparability of these studies might be restricted due to different compositions of the enzyme assays and different substrate analogues. Furthermore, activities determined with such artificial substrates do not allow direct inferences about *in situ* activities, but only enable a relative comparison between different samples or conditions.

### Responses of the microbial communities to plant polymer addition

**Fig 2:**
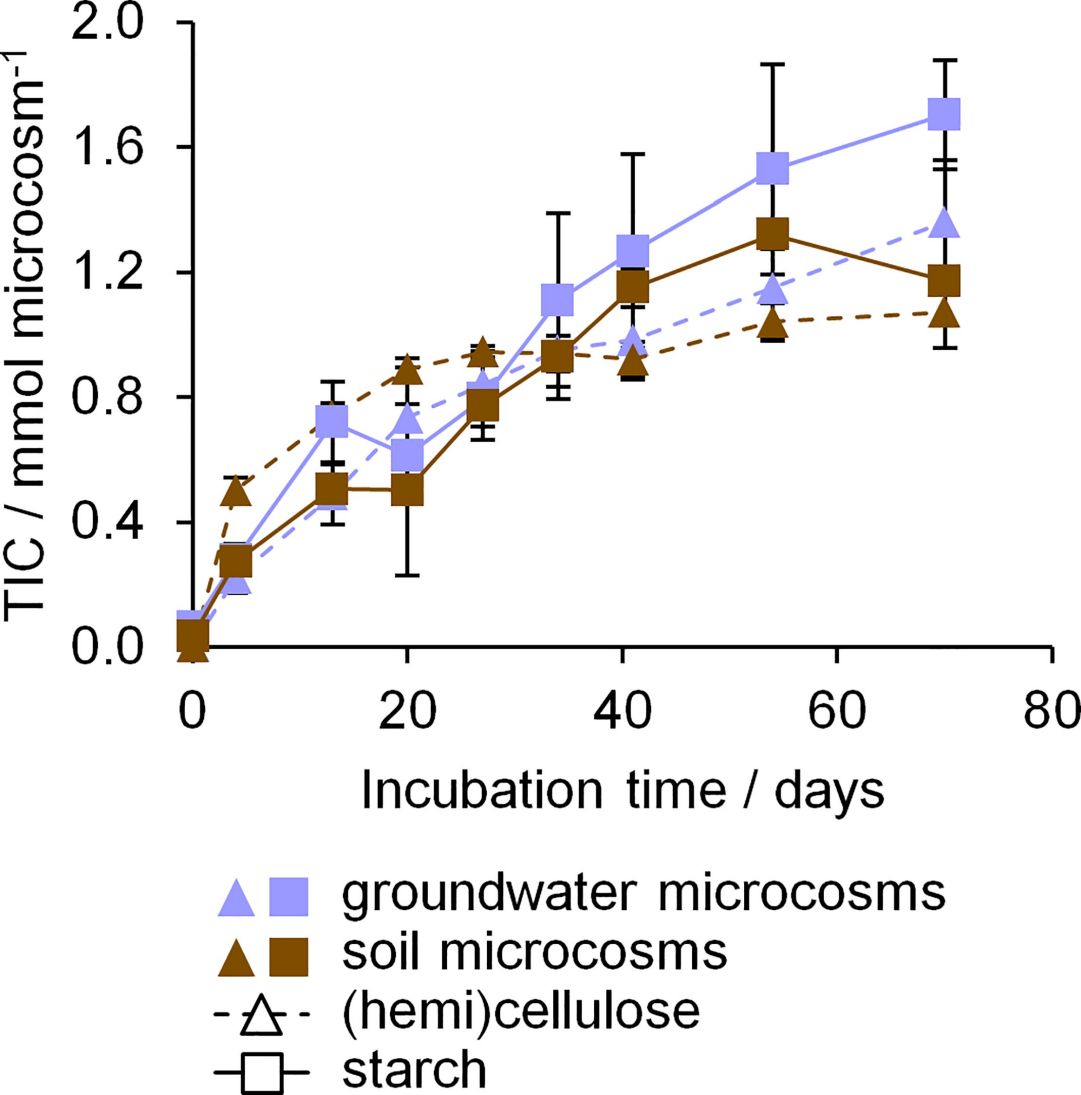
Total inorganic carbon (TIC) in groundwater and soil microcosms during incubation. Average values of five experimental replicates are given (3 with ^13^C polymers, 2 with unlabelled polymers). Error bars show standard deviation. Blue symbols, groundwater microcosms; brown symbols, soil microcosms. Triangles, microcosms with cellulose and hemicellulose; squares, microcosms with starch.

To follow the microbial utilization of plant biopolymers and investigate the microbial response during growth on plant polymers, we employed DNA-based stable isotope probing (SIP). Oxic microcosms containing filter pieces with groundwater biomass or soil were incubated with either ^13^C labelled starch or a mix of ^13^C labelled cellulose and hemicellulose. Additional microcosms with unlabelled biopolymers and without biopolymers were treated equally as controls. The oxidation of organic matter such as the added plant biopolymers to CO_2_ should lead to an increase of total inorganic carbon (TIC) in the microcosms. Hence, we monitored the TIC concentrations as proxy for plant biopolymer degradation (Fig 2, S1 File). The TIC production observed in groundwater microcosms was in the same range to that in soil microcosms. This was surprising, as soil microcosms contained two orders of magnitude more microorganisms and had three orders of magnitude higher enzyme activities at the start of the incubation compared to groundwater microcosms (S1 Table). Furthermore, no lag phase in TIC production was observed within the time scale of sampling. Between 1.36 and 1.70 mmol of TIC per groundwater microcosm were produced, compared to 1.07 to 1.32 mmol per soil microcosm. In the controls without biopolymers, no TIC production was observed in groundwater microcosms, but the background respiration in soil microcosms reached 50 to 65% of the TIC produced in microcosms with biopolymers (S1 Fig). Hence, while in groundwater microcosms, plant polymer addition increased TIC production from basically zero to 1.53 ± 0.24 mmol over the 70 days of incubation, in soil microcosms, only an increase of ~0.43 mmol ± 0.17 mmol (from 0.69 ± 0.06 mmol to 1.12 ± 0.16 mmol) occurred. The addition of biopolymers to groundwater created a distinctly stronger response than for soil. This is likely caused by the high amount of native carbon in the soil samples used, reported with up to 26.2 ± 0.77 g kg^-1^ in the top 10 cm [31]. The degradation of this native carbon would lead to the high TIC production observed in the microcosms without biopolymer addition. In contrast, the groundwater investigated contains only 1.9 ± 1.5 mg L^-1^ DOC [27]. Hence, the unspecific microbial activity (i.e. degradation of the native carbon instead of the biopolymers) was higher in soil than in groundwater microcosms. The produced TIC in both groundwater and soil microcosms corresponded to 20 to 31% of the total amount of biopolymer carbon added (5.55 mmol). Higher amounts of 38 to 61% produced TIC were previously reported during oxic incubation of soils with cellulose, cellobiose, and glucose [17]. Near the end of our incubations, oxygen in both groundwater and soil microcosms was likely depleted. Under anoxic conditions, the biopolymers can be utilized by fermenting organisms, which would lead to an incomplete oxidation. This can explain both the reduced rate of TIC increase near the end of the incubation time as well as the lower total amount of TIC produced. A decrease of pH due to acidic fermentation products, however, was not observed in groundwater microcosms, potentially because of buffering effects e.g. from the high bicarbonate concentrations. When comparing between types of plant polymers added, groundwater microcosms with (hemi)cellulose showed a slightly lower increase of TIC than those with starch (1.36 ± 0.18 mmol vs. 1.70 ± 0.15 mmol, *p* < 0.05, *t*-test). This might be caused by the better accessibility of starch for biodegradation due to the presence of refractory crystalline regions in (hemi)cellulose that can delay initial dissolution and hence utilization [7].

**Fig 3:**
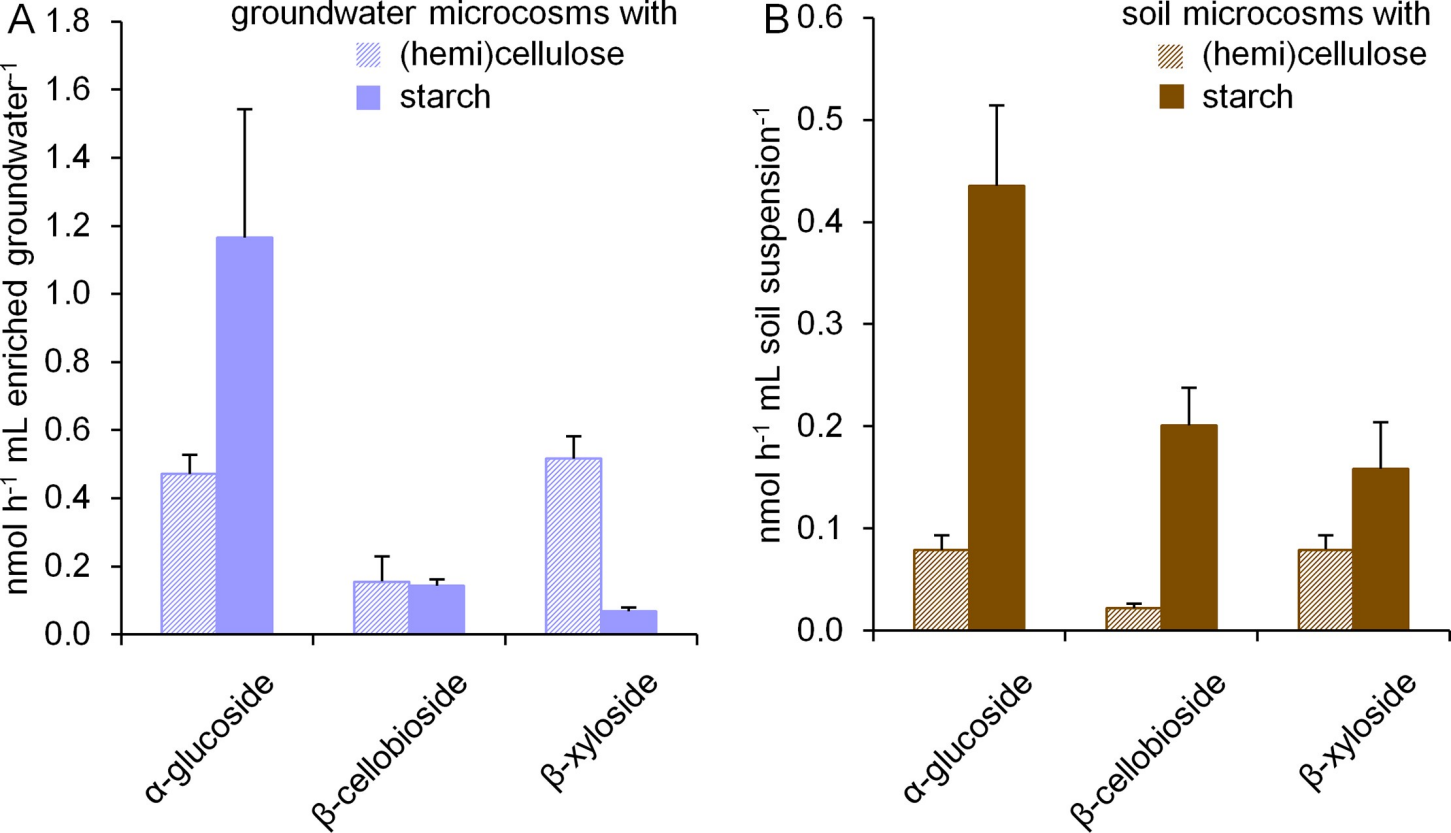
Enzyme activities in groundwater and soil microcosms after 70 days of incubation with addition of plant polymers. Activities on α-glucoside, β-cellobioside and β-xyloside are shown (A) in groundwater microcosms expressed per mL enriched groundwater and (B) in soil microcosms expressed per mL soil suspension. Values represent averages of enzyme activities in microcosms with (hemi)cellulose (striped bars, 3 technical replicates) and in microcosms with starch (filled bars, 3 technical replicates), and error bars depict standard deviations.

At the end of incubation, again enzyme activities were determined in microcosms with ^13^C labelled substrates to assess the response of the microbial community to the respective biopolymers. We found that groundwater and soil microcosms responded in the same way to the addition of biopolymers (Fig 3). After incubation with starch, the highest activities were observed for degradation of α-glucoside, followed by β-cellobioside and β-xyloside, which demonstrated the adaption of the microbial community to starch utilization. After incubation with (hemi)cellulose, almost equal activities for β-xyloside and α-glucoside degradation, and a slightly lower activity for β-cellobioside degradation were detected. Before incubation, α-glucoside degradation activities were one order of magnitude higher than the activities for both other substrates. Hence, our observations in microcosms incubated with (hemi)cellulose indicated a relative increase of activity for β-cellobioside and β-xyloside degradation. Enzymes degrading both substrates, such as cellobiohydrolase, β-glucosidases and xylanases, are often coproduced by the same organisms, and moreover exhibit substrate cross-specificity [7, 15]. The presence of both cellulose and hemicellulose was described to have a synergistic effect on xylosidase activity [43], which might explain the higher activity on β-xyloside after incubation with (hemi)cellulose. A preferred utilization of hemicellulose compared to cellulose in the microcosms might be plausible as well, as hemicellulose is less crystalline and thus more easily accessible [15]. Furthermore, soil microcosms supplemented with starch exhibited higher enzyme activities than those with (hemi)cellulose, a behavior not observed in groundwater microcosms. The higher amount of native organic carbon in the soil might allow a higher diversity of metabolic processes to occur, hence leading to a more complex response of the soil microbiome to priming with starch as an easily available carbon source, including a higher coproduction of plant polymer degrading enzymes targeting different substrates.

Our incubation experiments revealed that the groundwater microbiome in the Hainich CZE responded to addition of different biopolymers in a way highly similar to the soil microbiome. Despite a 100-fold lower number of cells at the start of incubation, plant polymer addition increased TIC production to levels that exceeded those observed in soil microcosms, with no apparent lag phase. Thus, the groundwater microbiome can confer analogous functions of plant polymer degradation as the soil microbiome. As the composition of the microbial community in groundwater is rather different from soil, we determined in the next step if distinct microbial taxa were involved in this function.

### Identification of key prokaryotic organisms involved in biopolymer degradation

**Fig 4:**
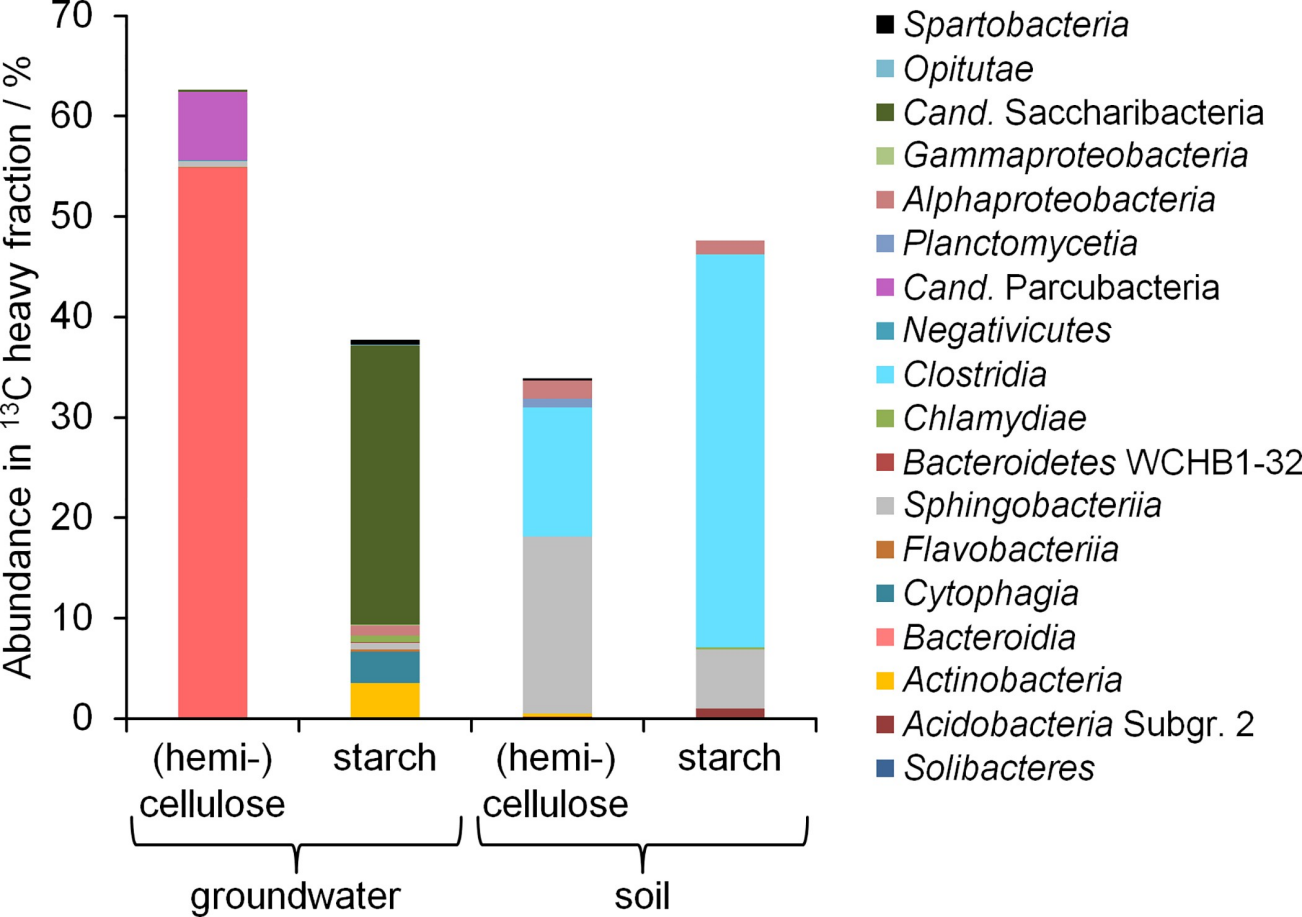
Taxonomic composition of the ^13^C labelled microbial communities. Relative abundance of class level taxonomic groups in the heavy DNA fraction of DNA from ^13^C labelled microcosms is shown. For each treatment, DNA samples of ^13^C triplicates were combined before ultracentrifugation and Illumina MiSeq amplicon sequencing of bacterial 16S rRNA genes. Only OTUs found to be significantly enriched in the heavy fraction of the ^13^C sample compared to the ^12^C sample are shown.

The utilization of ^13^C labelled plant polymers as carbon source will lead to the incorporation of ^13^C into the biomolecules of the microorganisms involved. We performed DNA-SIP for elucidating the identity of these microbial plant polymer-degraders, to answer the question if distinct microorganisms were stimulated by biopolymer addition in soil and groundwater. We were able to identify microbial groups significantly enriched (p < 0.05, Breslow-Day test for homogeneity of Odds Ratios [44]) in the heavy DNA fraction of ^13^C labelled samples from both groundwater and soil microcosm (S2 Table), demonstrating the utilization of plant polymers as carbon source by the groundwater microbial community. The labelled bacterial taxa observed included members of families well known for their ability to degrade plant polymers via extracellular enzymes, such as *Flavobacteriaceae* [45, 46], *Chitinophagaceae* [47], *Verrucomicrobia* [48], *Clostridiaceae* [49, 50], and *Ruminococcaceae* [51]. The activity of anaerobic bacteria, e.g. from the latter two families, might have been enabled by the depletion of oxygen in the microcosms during incubation. The most abundant labelled microorganisms identified differed between groundwater and soil microcosms (Fig 4). For groundwater, *Bacteroidia* and *Candidatus (Cand*.*)* Parcubacteria were dominant in (hemi)cellulose microcosms, while starch microcosms mostly comprised *Cand*. Saccharibacteria, *Cytophagia*, and *Actinobacteria*. For soil, *Clostridia*, *Sphingobacteriia* and *Alphaproteobacteria* were most abundant in the ^13^C heavy fractions from both (hemi)cellulose and starch microcosms. *Alphaproteobacteria* are the second most abundant taxonomic group in native soils of the Hainich forest (20.5 ± 0.4%), following the phylum *Acidobacteria*, as previously described [31]. *Clostridia* and *Sphingobacteriia*, however, were found to be only present in low abundance (< 1%) [31].

**Fig 5:**
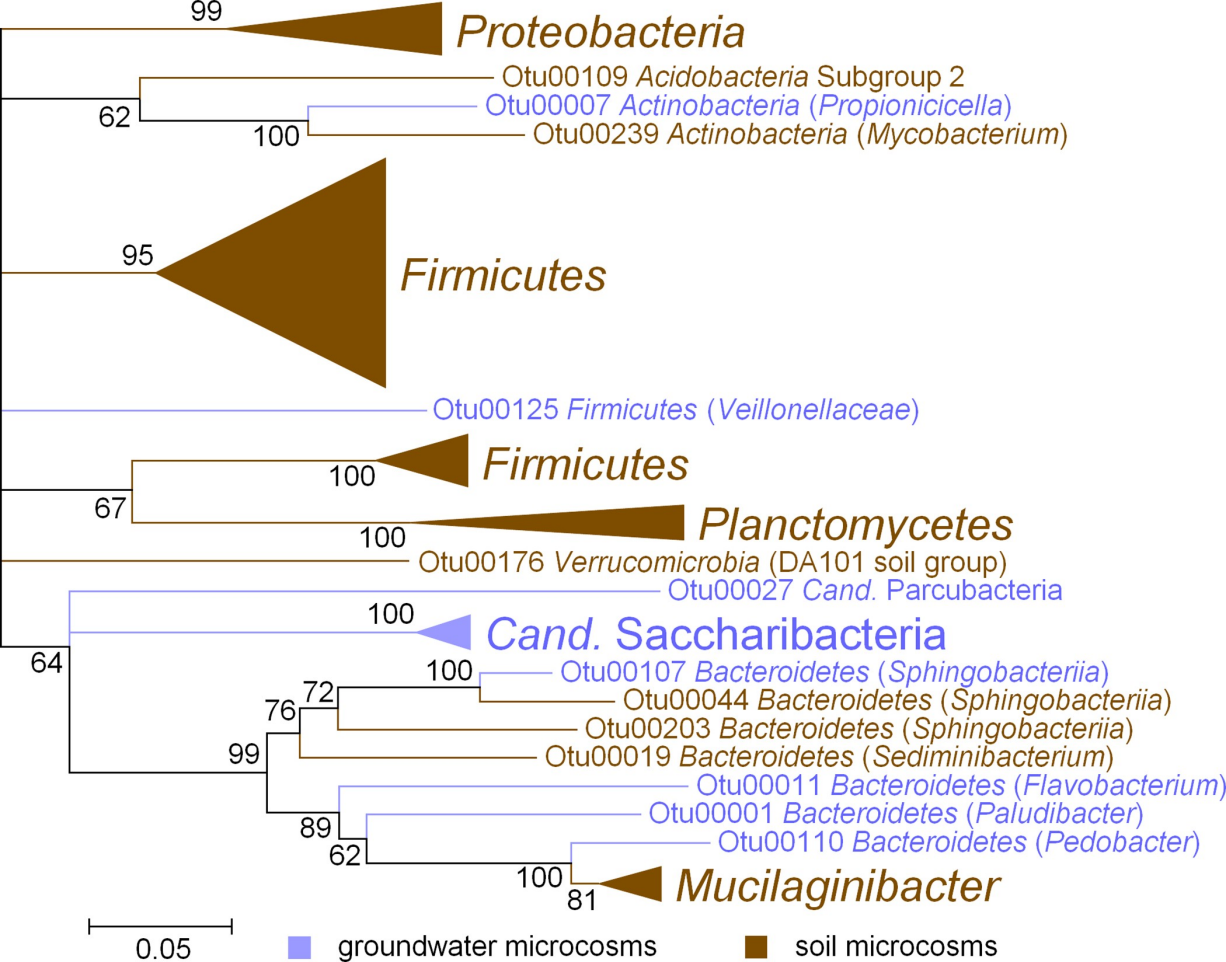
Phylogenetic relationship between ^13^C labelled OTUs from microcosms incubated with ^13^C (hemi)cellulose. Sequence data was derived from Illumina MiSeq amplicon sequencing of 16S rRNA genes using primers Bakt_341F and Bakt_785R. The tree was constructed using the neighbor-joining method for clustering and the maximum composite likelihood method for computing evolutionary distances. Numbers at branches, bootstrap values of 500 replicates. Scale bar, 1 nucleotide substitution per 20 nucleotides. Blue, OTUs from groundwater microcosms; brown, OTUs from soil microcosms.

**Fig 6:**
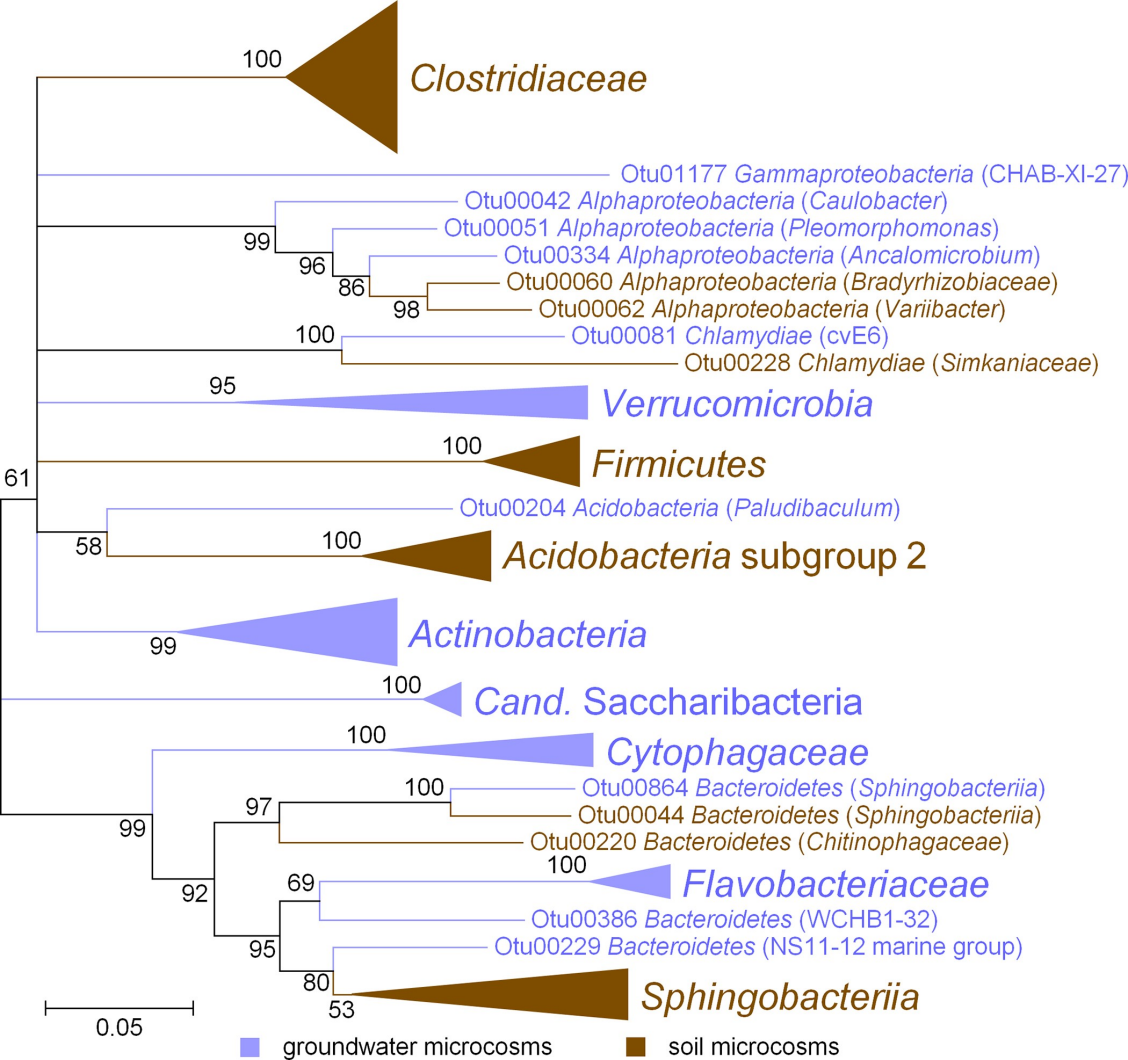
Phylogenetic relationship between ^13^C labelled OTUs from microcosms incubated with ^13^C starch. Sequence data was derived from Illumina MiSeq amplicon sequencing of 16S rRNA genes using primers Bakt_341F and Bakt_785R. The tree was constructed using the neighbor-joining method for clustering and the maximum composite likelihood method for computing evolutionary distances. Numbers at branches, bootstrap values of 500 replicates. Scale bar, 1 nucleotide substitution per 20 nucleotides. Blue, OTUs from groundwater microcosms; brown, OTUs from soil microcosms.

Less abundant microbial taxa were found in both groundwater and soil microcosms to be significantly enriched in the ^13^C heavy fractions. However, these taxa were clearly separated at higher taxonomic levels (Fig 5, Fig 6). For example, *Actinobacteria* were involved in (hemi)cellulose degradation both in groundwater and soil microcosms, but were related to *Propionicicella* (groundwater) or *Mycobacterium* (soil) on the genus level. Not one single OTU was found to be significantly enriched in both the ^13^C heavy fractions of soil and groundwater microcosms.

Our results demonstrated that despite the analogous functional patterns for plant polymer degradation observed, completely different microbial taxa were involved in these processes in groundwater and soil microcosms, suggesting a high level of functional redundancy. Interestingly, in groundwater microcosms especially members of the candidate superphylum *Cand*. Patescibacteria, the *Cand*. Parcubacteria and *Cand*. Saccharibacteria, were found to be active. Microorganisms affiliated to *Cand*. Patescibacteria are highly abundant in groundwater of the Hainich CZE, often making up more than 50% of the groundwater microbiome [28, 52]. *Cand*. Saccharibacteria are widespread in nature, from soil, sediment, and wastewater habitats to animals [53]. Their ability to degrade polysaccharides via extracellular enzymes has been deduced from genomic analyses [54]. Likewise, some members of the highly diverse superphylum *Cand*. Parcubacteria were reported to have the genomic potential for cellulose utilization [55, 56].

Members of the *Cand*. Patescibacteria are in general characterized by ultra-small cell sizes below 1 μm and reduced genomes of typically around 1 Mbp [18, 54]. A small genome allows lower maintenance energy costs [57], while reduced cell sizes increase the surface to volume ratio, improving the uptake of nutrients [58]. Such properties might be particular adaptations to life in the oligotrophic groundwater and be responsible for the success of *Cand*. Patescibacteria under these conditions. Furthermore, genomic characterization of *Cand*. Patescibacteria revealed the absence of several essential biosynthetic pathways [56, 59, 60], and it was hypothesized that these organisms depend on the uptake of certain amino acids or nucleotides from other bacteria. This would indicate a symbiotic or syntrophic lifestyle of *Cand*. Patescibacteria [59, 60]. Their interaction partners in the groundwater are yet unknown.

Genes encoding glucoside hydrolases involved in the degradation of starch, cellulose and hemicellulose are almost universally present in the *Cand*. Patescibacteria genomes known so far [56]. Exhibiting a fermentative lifestyle, these organisms might degrade the plant polysaccharides in our SIP incubations and release labile fermentation products that can be used by their partner organisms. Alternatively, the *Cand*. Patescibacteria might be labeled by cross-feeding. This can occur either through their prokaryotic or eukaryotic partners (the latter being present with 10^2^ to 10^3^ cells mL^-1^ in the groundwater of the Hainich CZE [61]), or by uptake of necromass from other microorganisms. The DNA-SIP approach used here is not able to differentiate between organisms directly using the labelled substrate and cross-feeding organisms [62]. Hence, it cannot be excluded that a part of the ^13^C enriched OTUs were actually utilizing metabolic products or residues of other organisms. The high abundance of *Cand*. Patescibacteria among the labelled organisms can be the consequence of both a direct involvement in plant polymer degradation as well as cross-feeding.

Interestingly, the stimulated microbial families detected by SIP in the groundwater microcosms typically make up less than 1% of the microbiome present in the aquifers of the Hainich CZE [1, 28, 52]. This is also true for the labelled OTUs of *Cand*. Parcubacteria and *Cand*. Saccharibacteria detected in the groundwater microcosms. In contrast, individual families of the active soil microbes detected by SIP make up 1 to 12% of the microbial community in the Hainich forest soils [31]. Hence, despite their low initial abundance, the organisms of the groundwater microbiome seemed to be poised to respond quickly to plant polysaccharide addition, and carried out the same ecological functions as the soil microbiome when it comes to plant polymer degradation, resulting in the analogous activity patterns observed. Unlike in soil microcosms, where similar microbial taxa were found to be involved in starch as well as (hemi)cellulose degradation, the taxa responding to these different plant polymers in groundwater were distinct, indicating niche differentiation due to specialization to a particular substrate. In soil, where the organic carbon content is orders of magnitude higher, selection might favor generalist microbes able to degrade different kinds of polymers instead.

### Conclusions

We demonstrated that the groundwater microbiome was able to perform an important ecosystem function like plant polymer degradation in a manner analogous to the soil microbiome investigated. Almost identical enzyme activity patterns and responses to plant polymer addition were observed. However, the organisms carrying out these functions in the groundwater were completely distinct from the responsible *Bacteria* in soil. Most notably, members of candidate phyla *Cand*. Parcubacteria and *Cand*. Saccharibacteria were enriched in the ^13^C heavy DNA fraction, indicating the assimilation of carbon from plant polymers by these organisms, either directly or via cross-feeding. Considering their high abundance, the involvement of candidate phyla in such processes implicates fundamental consequences for carbon cycling in the pristine groundwater of the Hainich CZE. The unique design of the Hainich CZE enables tracing the flow of groundwater from the recharge area on the summit of a hill, and hence allows unprecedented insights into pristine shallow aquifer systems. It remains open if the observations made here can be generalized to aquifers in different geological settings.

## Methods

### Sampling site and sampling

Groundwater samples were obtained in February 2015 from the Hainich Critical Zone Exploratory (CZE) located in Thuringia, Germany, accessing two aquifer assemblages in a thin-bedded carbonate-siliciclastic setting [8, 26]. Sampling permits were issued by the state environmental offices of Thuringia and the Hainich National Park Authority (Bad Langensalza, Germany). A volume of 5,000 L of groundwater from well H41 (51.1150842N 10.4479713E) was used. This well accesses the lower, oxic aquifer (Hainich Transect Lower Aquifer Assemblage—HTL) in the Trochitenkalk formation of the Upper Muschelkalk (Middle Triassic) in 48 m depth. Groundwater was pumped and filtered over a glass fiber filter with a pore size of 0.3 μm and 293 mm diameter (Sterlitech, Kent, WA, USA) using a submersible pump (Grundfos SQE5-70, Grundfos, Bjerringbro, Denmark) with a pump rate of 21 to 22 L min^-1^. Hydrogeochemical parameters in this groundwater well, including pH and the concentrations of DO, ammonium, DOC, and TIC, are monitored every four weeks as described [27]. In contrast to the methods used here, carbon sum parameters of the groundwater (DOC, TIC) are determined by high temperature catalytic oxidation (multi 18 N/C 2100S, AnalytikJena, Germany) [27].

Soil samples from three randomly chosen locations in the beech forest (*Fagus sylvatica*) around well H41, which closely resembles the surface of the main recharge area of the HTL aquifer assemblage, were obtained and treated individually as biological replicates. At each location, several hundred grams of the upper soil horizon (0–30 cm depth) were taken and stored in zip lock bags. The locations were at least 20–30 m away from each other to avoid spatial autocorrelation, and samples were taken at least 2 m away from the nearest trunk to minimize the influence of the rhizosphere or stem flow.

### Enzyme activity measurements

To measure the activity of plant polymer degrading enzymes in soil and groundwater samples, a fluorescence assay utilizing substrate analogues coupled to the fluorescent dye methylumbelliferon (MUF) was used. Sterile 10 mM stock solutions of the substrates 4-methylumbelliferyl-α-glucoside, 4-methylumbelliferyl-ß-cellobioside, and 4-methylumbelliferyl-ß-xyloside (Sigma-Aldrich, Taufkirchen, Germany) were prepared. MUF-α-glucoside was used to assess the activity of starch degrading α-glucosidase, MUF-ß-cellobioside and MUF-ß-xyloside were used to assess the activities of several cellulose and hemicellulose degrading enzymes such as cellobiohydrolases and xylosidases.

Soil samples were homogenized by suspending 1 g of soil in 50 mL of ddH_2_O. The three soil samples were treated individually as biological replicates. Groundwater samples were prepared by resuspending filter surface corresponding to 500 L of groundwater in 3 mL of ddH_2_O. Three filter pieces were treated individually as technical replicates. Activity assays were prepared in 96 well plates in triplicates, containing 50 μl sample, 50 μl 1 mM MUF substrate, and 100 μl 100 mM 2-(N-morpholino)ethanesulfonic acid (MES) buffer, pH 6. The assays were incubated at 30°C in a microplate reader (Microplate Reader Synergy H4, Bio-Tek Instruments Inc.). Fluorescence intensity was measured once per minute over 4 to 8 hours, using an excitation wavelength of 360 nm and a detection wavelength of around 450–460 nm. Concentrations of released MUF were calculated based on a standard curve with MUF concentrations of 0, 0.5, 1.0, 1.5, 2.0, 3.5, and 7.5 μM.

### SIP incubation experiments

To identify microorganisms assimilating carbon from plant biopolymers, soil and groundwater samples were incubated with ^13^C labelled plant polymers. Labelled starch, cellulose and hemicellulose (>97 atom % ^13^C) from maize (*Zea mays*) were obtained from IsoLife (Wageningen, The Netherlands). For soil samples, soil suspensions from 10 g of soil and 10 mL of autoclaved ddH_2_O were used for microcosm incubations. Microcosms of groundwater samples contained filter surface corresponding to 250 L of filtered groundwater (approximately 33 cm^2^) resuspended in 20 mL of unfiltered groundwater. As model plant polymers, either 150 mg starch or a mixture of 75 mg cellulose and 75 mg hemicellulose were added to each microcosm. Microcosms with ^13^C polymers were set up in triplicates and additional microcosms with ^12^C polymers were set up in duplicates. Incubation in 120 mL serum vials crimp sealed with butyl rubber stoppers was performed for 70 days on a rotational shaker with 280 rpm at 15°C in the dark. During incubation, TIC, pressure, and pH were monitored (S1 File). At the end of incubations, again enzyme activity measurements with undiluted samples from the microcosms were performed as described above.

### Extraction of DNA

Microcosm incubation was stopped after 70 days. The content of the microcosms was centrifuged at 9,000 xg for 10 min at room temperature in a Beckman GS-15R Centrifuge (Beckman Coulter GmbH, Krefeld, Germany) and the supernatant was removed. The pellet was stored at -20°C until extraction. The DNA extraction was performed with the RNA PowerSoil Total RNA Isolation Kit (MoBio, Carlsbad, CA, USA) and the additional RNA PowerSoil DNA Elution Accessory Kit (MoBio) following the manufacturer’s instructions. For extractions from environmental samples, 2 g of homogenized soil or filter surface corresponding to 150 L of filtered groundwater were used. The Quant-iT PicoGreen dsDNA assay (Thermo Fisher Scientific, Waltham, MA, USA) was used for DNA quantification, following the manufacturer’s instructions, in the microplate reader described above. The used wavelengths for excitation and emission were 480 nm and 520 nm, respectively.

### Quantitative polymerase chain reaction

To determine abundance of bacterial 16S rRNA genes in groundwater and soil samples, quantitative polymerase chain reaction (qPCR) was performed using the primer pair Bac8Fmod/Bac338EUB [63, 64]. Reactions were carried out according to cycling conditions and standards as previously described [65], using Maxima SYBR Green qPCR Master Mix (Thermo Fisher Scientific) on an Mx300P cycler (Agilent, Santa Clara, CA, USA). Groundwater samples were diluted 1:20, soil samples were measured in dilution series from 1:10 to 1:1000 due to possible inhibition effects of humic acids.

### DNA-SIP centrifugation and fractionation

Samples of DNA obtained from replicates were pooled for the ultracentrifugation. For each treatment, 5 μg of DNA were added to a mixture of 7.163 M cesium chloride (CsCl) and gradient buffer (0.1 M Tris, 0.1 M KCl, and 1 mM EDTA, pH 8) set to a final density of 1.725 g mL^−1^. Ultracentrifugation was carried out for 60 h at 20°C and 44,100 rpm. (~177,000 g) with vacuum, maximum acceleration, and no brake, using an NVT 90 rotor and a XL-70 Ultracentrifuge (Beckman Coulter). CsCl gradients were separated into 12 fractions of approximately 425 μl, and fraction density was calculated based on refractive indices obtained with an AR200 refractometer (Reichert technologies, Buffalo, NY, USA). DNA from all fractions was precipitated by adding 20 μg of glycogen and 900 μl of polyethylene glycol (PEG)-NaCl 6000 solution (30%/1.6 M), and left at room temperature for 1 h before centrifugation at 13,000 g for 30 min. DNA pellets were washed with 500 μl of 70% (v/v) ethanol, centrifuged for another 10 min, air-dried for 15 min and re-suspended in 50 μl of TE buffer (10 mM Tris-HCl, 1 mM EDTA). Heavy (^13^C) and light (^12^C) DNA samples of ^13^C incubations were selected for 16S rRNA gene amplicon sequencing based on density (1.725 and 1.700 g mL^−1^ for heavy and light fractions, respectively) according to Neufeld and colleagues [22]. For comparison, also the corresponding samples of ^12^C incubations were subjected to amplicon sequencing.

### Sequencing of 16S rRNA gene amplicons

Polymerase chain reactions using the light and heavy DNA fractions as template and targeting the V1 to V5 region of bacterial 16S rRNA genes were performed for 25 cycles with primer set 27F/907R (amplicon size approximately 900 bp) [66] using the following parameters: 10 min 95°C pre-denaturation; 95°C for 45 s, 55°C for 45 s, 72°C for 60 s; 10 min final elongation at 72°C. PCR products were sent for Illumina MiSeq sequencing (2x 300 bp paired end, LGC, Berlin, Germany) (S1 File). Raw sequence data was processed using mothur (www.mothur.org) and the mothur MiSeq SOP [67] as of 19th July 2016. Paired reads were combined, and sequences below 400 bp or above 429 bp as well as sequences with ambiguous bases or homopolymer runs above 8 bp were removed. Sequences were aligned to the SILVA reference database release SSU 119 [68] and sequences with differences of four bases or less were clustered. Chimera were removed with uchime using the GOLD database as reference [69]. Sequences were binned to OTUs with a 97% identity clustering threshold and OTUs were classified using the SILVA database described above.

### Determination of taxonomic groups from ^13^C enriched DNA

To assess which microbial taxa were enriched in the heavy DNA fraction from ^13^C incubations, abundances of taxonomic groups in light and heavy fractions were compared between the ^12^C and ^13^C incubation samples of each treatment. Due to the high complexity of the community profiles, containing up to 12,000 OTUs per sample, a statistical test was used to determine significantly enriched taxonomic groups. In this test, the Odds Ratios for a read to belong to a specific taxonomic group in the light or the heavy fraction sample were compared between ^12^C and ^13^C incubations using the Breslow-Day test for homogeneity of the Odds Ratios [44]. Odds Ratios (OR) were defined as

$$OR=(G_{H}/{nG}_{H})/(G_{L}/{nG}_{L})$$

with *G* or *nG* denoting the number of reads belonging or not belonging to a specific taxonomic group, and subscript *L* or *H* denoting light or heavy fraction sample. When comparing ^12^C and ^13^C sample, a higher Odds Ratio in the ^13^C sample states that the taxonomic group of concern is more enriched in the heavy fraction of the ^13^C sample, i.e. likely ^13^C labelled. A higher Odds Ratio in the ^12^C sample denotes that the taxonomic group of concern is less enriched in the heavy fraction of the ^13^C sample, and consequently more enriched in the light fraction, i.e. unlabelled. A “higher” enrichment in the light fraction of the ^13^C sample can occur as labelled taxonomic groups are not present in the light fraction anymore, so unlabelled taxa have a higher abundance. The method used to determine whether the Odds Ratios differ significantly in ^12^C and ^13^C sample has the advantage to not require any normalization or subsampling of the reads, thus the statistical power benefits from the total read number in every sample. The calculated *p*-values scale inversely with the difference in Odds Ratio between ^12^C and ^13^C sample, the percentage of reads assigned to a taxonomic group, and the total number of reads obtained for each sample. To control the probability of type I errors (false positives), the Holm-Bonferroni method [70] was used, ordering the *p*-values for all *n* OTUs of a treatment from lowest to highest, and testing significance of the *k*th OTU with a corrected significance level *α* as:

p(k)<α/(n+1−k)

### Accession numbers

Raw data from Illumina MiSeq sequencing of 16S rRNA gene amplicons have been deposited in the Sequence Read Archive (SRA) of NCBI (https://www.ncbi.nlm.nih.gov/sra) under accession numbers SRX3471030 to SRX3471045.

## Supporting information

S1 File Supplementary Methods. Additional information about monitoring of TIC and 16S rRNA gene amplicon sequencing analysis.
(PDF)

S1 Fig Total inorganic carbon (TIC) in groundwater and soil microcosms with (hemi)cellulose, starch or no added substrate. Average values of five experimental replicates are given for microcosms with (hemi)cellulose or starch, and of three experimental replicates from microcosms without added substrate. Error bars represent standard deviation.
(TIF)

S1 Table Bacterial 16S rRNA genes in groundwater and soil samples as well as microcosms. Given are average values and standard deviation. Gene copy numbers for enriched groundwater and soil samples have been determined by qPCR in triplicate reactions. Gene copy numbers for microcosms have been calculated based on the amount of enriched groundwater (250 L) or soil (10 g) used to in the microcosm setups.
(PDF)

S2 Table OTUs significantly enriched in the heavy DNA fraction of ^13^C microcosms after 70 days of incubation with ^13^C labelled (hemi)cellulose or starch. Taxonomic classification of the OTUs based on SILVA reference database release SSU 119 is shown on phylum and class level, as well as on the highest level up to genus where classification was possible. Levels of significance are *: p < 0.05, **: p < 0.01, and ***: p < 0.001, based on Breslow-Day test with Holm-Bonferroni correction.
(PDF)

## References

[1] Herrmann M, Rusznyak A, Akob DM, Schulze I, Opitz S, Totsche KU, et al Large fractions of CO_2_-fixing microorganisms in pristine limestone aquifers appear to be involved in the oxidation of reduced sulfur and nitrogen compounds. Appl Environ Microb. 2015;81(7):2384–94. 10.1128/AEM.03269-14 PMC4357952 25616797

[2] Nowak ME, Schwab VF, Lazar CS, Behrendt T, Kohlhepp B, Totsche KU, et al Carbon isotopes of dissolved inorganic carbon reflect utilization of different carbon sources by microbial communities in two limestone aquifer assemblages. Hydrol Earth Syst Sc. 2017;21(9):4283–300.

[3] Ghiorse WC, Wilson JT. Microbial ecology of the terrestrial subsurface. Adv Appl Microbiol. 1988;33:107–72. 3041739 10.1016/s0065-2164(08)70206-5

[4] Griebler C, Lueders T. Microbial biodiversity in groundwater ecosystems. Freshwater Biol. 2009;54(4):649–77.

[5] Madsen EL, Ghiorse WC. Groundwater microbiology: subsurface ecosystem processes In: Ford TE, editor. Aquatic microbiology—An ecological approach. Oxford, England: Blackwell Scientific Publication; 1993.

[6] Bond-Lamberty B, Thomson A. Temperature-associated increases in the global soil respiration record. Nature. 2010;464(7288):579–U132. 10.1038/nature08930 20336143

[7] Lynd LR, Weimer PJ, van Zyl WH, Pretorius IS. Microbial cellulose utilization: Fundamentals and biotechnology. Microbiol Mol Biol R. 2002;66(3):506–77. 10.1128/MMBR.66.3.506-577.2002 PMC120791 12209002

[8] Küsel K, Totsche KU, Trumbore SE, Lehmann R, Steinhäuser C, Herrmann M. How deep can surface signals be traced in the critical zone? Merging biodiversity with biogeochemistry research in a central German Muschelkalk landscape. Front Earth Sci. 2016;4:32.

[9] Bischoff S, Schwarz MT, Siemens J, Thieme L, Wilcke W, Michalzik B. Properties of dissolved and total organic matter in throughfall, stemflow and forest floor leachate of central European forests. Biogeosciences. 2015;12(9):2695–706.

[10] Dibbern D, Schmalwasser A, Lueders T, Totsche KU. Selective transport of plant root-associated bacterial populations in agricultural soils upon snowmelt. Soil Biol Biochem. 2014;69:187–96.

[11] Pronk M, Goldscheider N, Zopfi J, Zwahlen F. Percolation and particle transport in the unsaturated zone of a karst aquifer. Ground Water. 2009;47(3):361–9. 19462487 10.1111/j.1745-6584.2008.00509.x

[12] Baker MA, Valett HM, Dahm CN. Organic carbon supply and metabolism in a shallow groundwater ecosystem. Ecology. 2000;81(11):3133–48.

[13] Cooper KJ, Whitaker FF, Anesio AM, Naish M, Reynolds DM, Evans EL. Dissolved organic carbon transformations and microbial community response to variations in recharge waters in a shallow carbonate aquifer. Biogeochemistry. 2016;129(1–2):215–34.

[14] Hubalek V, Wu XF, Eiler A, Buck M, Heim C, Dopson M, et al Connectivity to the surface determines diversity patterns in subsurface aquifers of the Fennoscandian shield. ISME J. 2016;10(10):2447–58. 10.1038/ismej.2016.36 27022994 PMC5030689

[15] Warren RAJ. Microbial hydrolysis of polysaccharides. Annu Rev Microbiol. 1996;50:183–212. 10.1146/annurev.micro.50.1.183 8905079

[16] Jeffries TW. Biodegradation of lignin and hemicelluloses In: Ratledge C, editor. Biochemistry of microbial degradation. Dordrecht, Netherlands: Springer; 1994.

[17] Schellenberger S, Kolb S, Drake HL. Metabolic responses of novel cellulolytic and saccharolytic agricultural soil bacteria to oxygen. Environ Microbiol. 2010;12(4):845–61. 10.1111/j.1462-2920.2009.02128.x 20050868

[18] Brown CT, Hug LA, Thomas BC, Sharon I, Castelle CJ, Singh A, et al Unusual biology across a group comprising more than 15% of domain Bacteria. Nature. 2015;523(7559):208–11. Epub 2015/06/18. 10.1038/nature14486 26083755

[19] Rinke C, Schwientek P, Sczyrba A, Ivanova NN, Anderson IJ, Cheng JF, et al Insights into the phylogeny and coding potential of microbial dark matter. Nature. 2013;499(7459):431–7. 10.1038/nature12352 23851394

[20] Rappé MS, Giovannoni SJ. The uncultured microbial majority. Annu Rev Microbiol. 2003;57:369–94. 10.1146/annurev.micro.57.030502.090759 14527284

[21] Lazar CS, Stoll W, Lehmann R, Herrmann M, Schwab VF, Akob DM, et al Archaeal diversity and CO_2_ fixers in carbonate-/siliciclastic-rock groundwater ecosystems. Archaea. 2017. 10.1155/2017/2136287 PMC5485487 28694737

[22] Neufeld JD, Vohra J, Dumont MG, Lueders T, Manefield M, Friedrich MW, et al DNA stable-isotope probing. Nat Protoc. 2007;2(4):860–6. 10.1038/nprot.2007.109 17446886

[23] Youngblut ND, Barnett SE, Buckley DH. SIPSim: A modeling toolkit to predict accuracy and aid design of DNA-SIP experiments. Front Microbiol. 2018;9:507 10.3389/fmicb.2018.00507 29643843 PMC5882788

[24] Hungate BA, Mau RL, Schwartz E, Caporaso JG, Dijkstra P, van Gestel N, et al Quantitative microbial ecology through stable isotope probing. Appl Environ Microb. 2015;81(21):7570–81. 10.1128/AEM.02280-15 PMC4592864 26296731

[25] Pepe-Ranney C, Campbell AN, Koechli CN, Berthrong S, Buckley DH. Unearthing the ecology of soil microorganisms using a high resolution DNA-SIP approach to explore cellulose and xylose metabolism in soil. Front Microbiol. 2016;7:703 10.3389/fmicb.2016.00703 27242725 PMC4867679

[26] Kohlhepp B, Lehmann R, Seeber P, Küsel K, Trumbore SE, Totsche KU. Aquifer configuration and geostructural links control the groundwater quality in thin-bedded carbonate-siliciclastic alternations of the Hainich CZE, central Germany. HESSD. 2017;21:6091–116.

[27] Kohlhepp B, Lehmann R, Seeber P, Küsel K, Trumbore SE, Totsche KU. Pedological and hydrogeological setting and subsurface flow structure of the carbonate-rock CZE Hainich in western Thuringia, Germany. HESSD. 2016.

[28] Schwab VF, Herrmann M, Roth VN, Gleixner G, Lehmann R, Pohnert G, et al Functional diversity of microbial communities in pristine aquifers inferred by PLFA- and sequencing -based approaches. Biogeosciences. 2016.

[29] Opitz S, Küsel K, Spott O, Totsche KU, Herrmann M. Oxygen availability and distance to surface environments determine community composition and abundance of ammonia-oxidizing prokaroytes in two superimposed pristine limestone aquifers in the Hainich region, Germany. FEMS Microbiol Ecol. 2014;90(1):39–53. 10.1111/1574-6941.12370 24953994

[30] Taubert M, Stöckel S, Geesink P, Girnus S, Jehmlich N, Von Bergen M, et al Tracking active groundwater microbes with D_2_O labeling to understand their ecosystem function. Environ Microbiol. 2017. 10.1111/1462-2920.14010 29194923

[31] Nacke H, Goldmann K, Schöning I, Pfeiffer B, Kaiser K, Castillo-Villamizar GA, et al Fine spatial scale variation of soil microbial communities under European beech and Norway spruce. Front Microbiol. 2016;7:2067 Epub 2017/01/10. 10.3389/fmicb.2016.02067 28066384 PMC5177625

[32] Pritsch K, Raidl S, Marksteiner E, Blaschke H, Agerer R, Schloter M, et al A rapid and highly sensitive method for measuring enzyme activities in single mycorrhizal tips using 4-methylumbelliferone-labelled fluorogenic substrates in a microplate system. J Microbiol Meth. 2004;58(2):233–41. 10.1016/j.mimet.2004.04.001 15234521

[33] Palmroth MRT, Münster U, Pichtel J, Puhakka JA. Metabolic responses of microbiota to diesel fuel addition in vegetated soil. Biodegradation. 2005;16(1):91–101. 15727158 10.1007/s10531-004-0626-y

[34] Badiane NNY, Chotte JL, Pate E, Masse D, Rouland C. Use of soil enzyme activities to monitor soil quality in natural and improved fallows in semi-arid tropical regions. Appl Soil Ecol. 2001;18(3):229–38.

[35] Acosta-Martinez V, Tabatabai MA. Enzyme activities in a limed agricultural soil. Biol Fert Soils. 2000;31(1):85–91.

[36] Chen RR, Blagodatskaya E, Senbayram M, Blagodatsky S, Myachina O, Dittert K, et al Decomposition of biogas residues in soil and their effects on microbial growth kinetics and enzyme activities. Biomass Bioenerg. 2012;45:221–9.

[37] Marx MC, Kandeler E, Wood M, Wermbter N, Jarvis SC. Exploring the enzymatic landscape: distribution and kinetics of hydrolytic enzymes in soil particle-size fractions. Soil Biol Biochem. 2005;37(1):35–48.

[38] Kolehmainen RE, Korpela JP, Münster U, Puhakka JA, Tuovinen OH. Extracellular enzyme activities and nutrient availability during artificial groundwater recharge. Water Res. 2009;43(2):405–16. 10.1016/j.watres.2008.10.048 19028394

[39] Stoddard SF, Smith BJ, Hein R, Roller BRK, Schmidt TM. rrnDB: improved tools for interpreting rRNA gene abundance in bacteria and archaea and a new foundation for future development. Nucleic Acids Res. 2015;43(D1):D593–D8. 25414355 10.1093/nar/gku1201 PMC4383981

[40] Cairns JRK, Esen A. beta-Glucosidases. Cell Mol Life Sci. 2010;67(20):3389–405. 10.1007/s00018-010-0399-2 20490603 PMC11115901

[41] Schütz K, Kandeler E, Nagel P, Scheu S, Ruess L. Functional microbial community response to nutrient pulses by artificial groundwater recharge practice in surface soils and subsoils. FEMS Microbiol Ecol. 2010;72(3):445–55. 10.1111/j.1574-6941.2010.00855.x 20557572

[42] Clinton SM, Edwards RT, Findlay SEG. Exoenzyme activities as indicators of dissolved organic matter composition in the hyporheic zone of a floodplain river. Freshwater Biol. 2010;55(8):1603–15.

[43] Pérez-Avalos O, Ponce-Noyola T, Magana-Plaza I, delaTorre M. Induction of xylanase and beta-xylosidase in *Cellulomonas flavigena* growing on different carbon sources. Appl Microbiol Biot. 1996;46(4):405–9.

[44] Liu I. Breslow–Day Test. Encyclopedia of Biostatistics. 2005.

[45] McBride MJ, Xie G, Martens EC, Lapidus A, Henrissat B, Rhodes RG, et al Novel features of the polysaccharide-digesting gliding bacterium *Flavobacterium johnsoniae* as revealed by genome sequence analysis. Appl Environ Microb. 2009;75(21):6864–75. 10.1128/AEM.01495-09 PMC2772454 19717629

[46] Okamoto K, Nakano H, Yatake T, Kiso T, Kitahata S. Purification and some properties of a beta-glucosidase from *Flavobacterium johnsonae*. Biosci Biotech Bioch. 2000;64(2):333–40. 10.1271/bbb.64.333 10737190

[47] Bailey VL, Fansler SJ, Stegen JC, Mccue LA. Linking microbial community structure to beta-glucosidic function in soil aggregates. ISME J. 2013;7(10):2044–53. 10.1038/ismej.2013.87 23719152 PMC3965315

[48] Yarwood S, Brewer E, Yarwood R, Lajtha K, Myrold D. Soil microbe active community composition and capability of responding to litter addition after 12 years of no inputs. Appl Environ Microb. 2013;79(4):1385–92. 10.1128/AEM.03181-12 PMC3568599 23263952

[49] Zhou YZ, Pope PB, Li SC, Wen B, Tan FJ, Cheng S, et al Omics-based interpretation of synergism in a soil-derived cellulose-degrading microbial community. Sci Rep. 2014;4:5288 10.1038/srep05288 24924356 PMC5381534

[50] Lee SF, Forsberg CW, Gibbins LN. Cellulolytic activity of *Clostridium acetobutylicum*. Appl Environ Microb. 1985;50(2):220–8. 10.1128/aem.50.2.220-228.1985 PMC238607 16346847

[51] Flint HJ, Scott KP, Duncan SH, Louis P, Forano E. Microbial degradation of complex carbohydrates in the gut. Gut microbes. 2012;3(4):289–306. Epub 2012/05/11. 10.4161/gmic.19897 22572875 PMC3463488

[52] Kumar S, Herrmann M, Thamdrup B, Schwab VF, Geesink P, Trumbore SE, et al Nitrogen loss from pristine carbonate-rock aquifers of the Hainich Critical Zone Exploratory (Germany) is primarily driven by chemolithoautotrophic anammox processes. Front Microbiol. 2017;8:1951 10.3389/fmicb.2017.01951 29067012 PMC5641322

[53] Ferrari B, Winsley T, Ji M, Neilan B. Insights into the distribution and abundance of the ubiquitous *Candidatus* Saccharibacteria phylum following tag pyrosequencing. Sci Rep. 2014;4. 10.1038/srep03957 PMC5379237 24492458

[54] Albertsen M, Hugenholtz P, Skarshewski A, Nielsen KL, Tyson GW, Nielsen PH. Genome sequences of rare, uncultured bacteria obtained by differential coverage binning of multiple metagenomes. Nat Biotechnol. 2013;31(6):533–8. 10.1038/nbt.2579 23707974

[55] Wrighton KC, Castelle CJ, Wilkins MJ, Hug LA, Sharon I, Thomas BC, et al Metabolic interdependencies between phylogenetically novel fermenters and respiratory organisms in an unconfined aquifer. ISME J. 2014;8(7):1452–63. 10.1038/ismej.2013.249 24621521 PMC4069391

[56] Danczak RE, Johnston MD, Kenah C, Slattery M, Wrighton KC, Wilkins MJ. Members of the Candidate Phyla Radiation are functionally differentiated by carbon- and nitrogen-cycling capabilities. Microbiome. 2017;5(1):112 10.1186/s40168-017-0331-1 28865481 PMC5581439

[57] Giovannoni SJ, Thrash JC, Temperton B. Implications of streamlining theory for microbial ecology. ISME J. 2014;8(8):1553–65. 10.1038/ismej.2014.60 24739623 PMC4817614

[58] Sowell SM, Wilhelm LJ, Norbeck AD, Lipton MS, Nicora CD, Barofsky DF, et al Transport functions dominate the SAR11 metaproteome at low-nutrient extremes in the Sargasso Sea. ISME J. 2009;3(1):93–105. 10.1038/ismej.2008.83 18769456

[59] Anantharaman K, Brown CT, Burstein D, Castelle CJ, Probst AJ, Thomas BC, et al Analysis of five complete genome sequences for members of the class Peribacteria in the recently recognized Peregrinibacteria bacterial phylum. Peerj. 2016;4:e1607 10.7717/peerj.1607 26844018 PMC4736985

[60] Nelson WC, Stegen JC. The reduced genomes of Parcubacteria (OD1) contain signatures of a symbiotic lifestyle. Front Microbiol. 2015;6:713 10.3389/fmicb.2015.00713 26257709 PMC4508563

[61] Risse-Buhl U, Herrmann M, Lange P, Akob DM, Pizani N, Schönborn W, et al Phagotrophic protist diversity in the groundwater of a karstified aquifer—morphological and molecular analysis. J Eukaryot Microbiol. 2013;60(5):467–79. 10.1111/jeu.12054 23808986

[62] Dumont MG, Murrell JC. Stable isotope probing—linking microbial identity to function. Nat Rev Microbiol. 2005;3(6):499–504. 10.1038/nrmicro1162 15886694

[63] Daims H, Brühl A, Amann R, Schleifer KH, Wagner M. The domain-specific probe EUB338 is insufficient for the detection of all Bacteria: Development and evaluation of a more comprehensive probe set. Syst Appl Microbiol. 1999;22(3):434–44. 10.1016/S0723-2020(99)80053-8 10553296

[64] Loy A, Lehner A, Lee N, Adamczyk J, Meier H, Ernst J, et al Oligonucleotide microarray for 16S rRNA gene-based detection of all recognized lineages of sulfate-reducing prokaryotes in the environment. Appl Environ Microb. 2002;68(10):5064–81. 10.1128/AEM.68.10.5064-5081.2002 PMC126405 12324358

[65] Herrmann M, Hädrich A, Küsel K. Predominance of thaumarchaeal ammonia oxidizer abundance and transcriptional activity in an acidic fen. Environ Microbiol. 2012;14(11):3013–25. 10.1111/j.1462-2920.2012.02882.x 23016896

[66] Lane DJ. 16S/23S rRNA sequencing In: Stackebrandt E, Goodfellow M, editors. Nucleic acid techniques in bacterial systematics. New York, NY: John Wiley and Sons; 1991 p. 115–75.

[67] Kozich JJ, Westcott SL, Baxter NT, Highlander SK, Schloss PD. Development of a dual-index sequencing strategy and curation pipeline for analyzing amplicon sequence data on the MiSeq Illumina sequencing platform. Appl Environ Microb. 2013;79(17):5112–20. 10.1128/AEM.01043-13 PMC3753973 23793624

[68] Quast C, Pruesse E, Yilmaz P, Gerken J, Schweer T, Yarza P, et al The SILVA ribosomal RNA gene database project: improved data processing and web-based tools. Nucleic Acids Res. 2013;41(D1):D590–D6. 23193283 10.1093/nar/gks1219 PMC3531112

[69] Reddy TBK, Thomas AD, Stamatis D, Bertsch J, Isbandi M, Jansson J, et al The Genomes OnLine Database (GOLD) v.5: a metadata management system based on a four level (meta)genome project classification. Nucleic Acids Res. 2015;43(D1):D1099–D106. 25348402 10.1093/nar/gku950 PMC4384021

[70] Holm S. A simple sequentially rejective multiple test procedure. Scand J Stat. 1979;6(2):65–70.

