# Supplementary material for: Divergent microbial communities in groundwater and overlying soils exhibit functional redundancy for plant-polysaccharide degradation
Source: PLoS One. 2019 Mar 13;14(3):e0212937. doi: 10.1371/journal.pone.0212937 (PMC6415789; doi:10.1371/journal.pone.0212937)
Supplement: S1 File — Additional information about monitoring of TIC and 16S rRNA gene amplicon sequencing analysis. (PDF) [file pone.0212937.s001.pdf]

S1 SUPPORTING INFORMATION

Divergent microbial communities in groundwater and overlying soils exhibit functional redundancy for plant-polysaccharide degradation

Martin Taubert[1,*], Jan Stähly[1], Steffen Kolb[1,2], Kirsten Küsel[1,3]

[1]Aquatic Geomicrobiology, Institute of Biodiversity, Friedrich Schiller University Jena, Jena, Germany

[2]Microbial Biogeochemistry, RA Landscape Processes, Leibniz Centre for Agricultural Landscape Research (ZALF), Müncheberg, Germany

[3]German Centre for Integrative Biodiversity Research (iDiv) Halle-Jena-Leipzig, Leipzig, Germany

[*]Corresponding author

E-mail: martin.taubert@uni-jena.de (MT)

Running title: Plant polysaccharide degradation in groundwater and soil

Materials and Methods

Monitoring of physicochemical parameters in microcosms during SIP incubation

During incubation, the concentration of total inorganic carbon (TIC) as well as pressure and pH were monitored every 1 to 2 weeks. Quantification of TIC was based on CO_2 partial pressure in the headspace of the incubations determined by GC measurements on a Hewlett Packard 5890 instrument (Hewlett Packard, Palo Alto, CA, USA) with a thermal conductivity detector, equipped with a Chromosorb 102 column (2 m x 1/8'', Alltech, Unterhaching, Germany). The injector temperature was set to 150 °C and the detector temperature to 175 °C. A volume of 100 µl from the headspace of the incubations was injected and separated with Helium as carrier gas at a pressure of 22 psi and 50 °C, CO_2 was detected after 1:35 min. Pressure in the incubations was measured with a portable digital pressure meter GMH 3111 (Greisinger electronic, Regenstauf, Germany) before taking samples for GC measurements, and pH was measured with a WTW Sentix Mic probe attached to a WTW pH 330 pH-meter (WTW electronic, Graz, Austria). Pressure and pH were used to calculate the concentrations of dissolved hydrogen carbonate for determination of TIC as previously described (1).

Bacterial 16S rRNA gene amplicon sequencing by LGC

Illumina MiSeq amplicon sequencing targeting bacterial 16S rRNA genes was performed on all samples using the primer combinations Bakt_785R/Bakt_341F (2). Illumina MiSeq amplicon sequencing was carried out by LGC Genomics GmbH (Berlin, Germany). The PCRs included about 5 ng of DNA extract, 15 pmol of each forward primer 341F 5'-NNNNNNNNNNTCCTACGGGNGGCWGCAG and reverse primer 785R 5'-

NNNNNNNNNNTGACTACHVGGGTATCTAAKCC in 20 uL volume of 1 x MyTaq buffer containing 1.5 units MyTaq DNA polymerase (Bioline) and 2 µl of BioStabII PCR Enhancer (Sigma). For each sample, the forward and reverse primers had the same 10-nt barcode sequence, resulting in an amplicon size of approximately 470 bp, including the V3 and V4 region of the bacterial 16S rRNA gene. PCRs were carried out for 30 cycles using the following parameters: 2 min 96 °C pre-denaturation; 96 °C for 15 s, 50 °C for 30 s, 70 °C for 90 s. DNA concentration of amplicons of interest was determined by gel electrophosesis . About 20 ng amplicon DNA of each sample were pooled for up to 48 samples carrying different barcodes. If needed PCRs showing low yields were further amplified for 5 cycles. The amplicon pools were purified with one volume AMPure XP beads (Agencourt) to remove primer dimer and other small mispriming products, followed by an additional purification on MinElute columns (Qiagen). About 100 ng of each purified amplicon pool DNA was used to construct Illumina libraries using the Ovation Rapid DR Multiplex System 1-96 (NuGEN). Illumina libraries were pooled and size selected by preparative gel electrophoresis. Sequencing was done on an Illumina MiSeq using V3 Chemistry (Illumina).

References

1. Küsel K, Drake HL. Effects of environmental parameters on the formation and turnover of acetate by forest soils. Appl Environ Microb. 1995;61(10):3667-75.

2. Herlemann DPR, Labrenz M, Jürgens K, Bertilsson S, Waniek JJ, Andersson AF. Transitions in bacterial communities along the 2000 km salinity gradient of the Baltic Sea. ISME J. 2011;5(10):1571-9.
