# Supplementary figures and images for: Divergent microbial communities in groundwater and overlying soils exhibit functional redundancy for plant-polysaccharide degradation
Source: PLoS One. 2019 Mar 13;14(3):e0212937. doi: 10.1371/journal.pone.0212937 (PMC6415789; doi:10.1371/journal.pone.0212937)

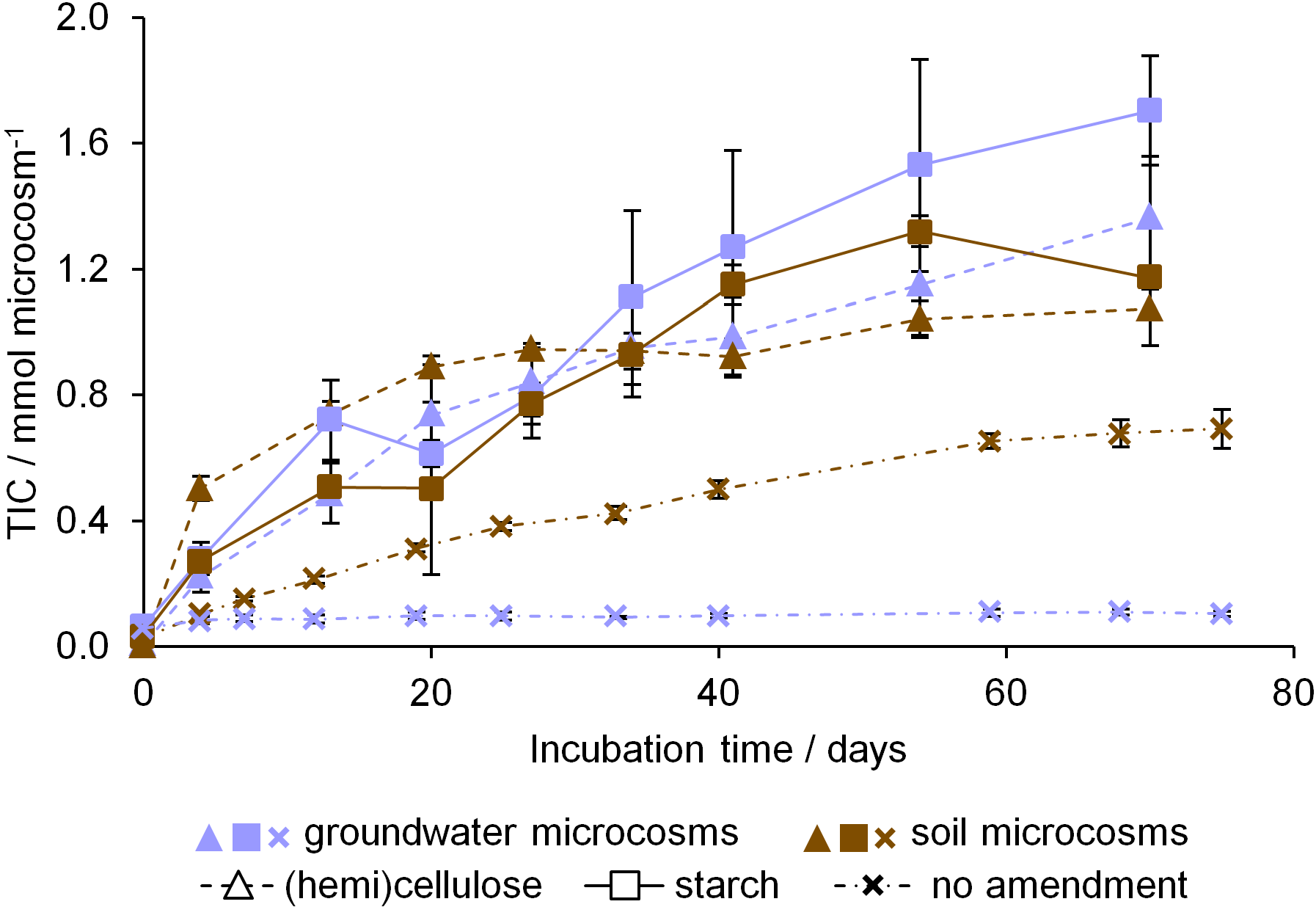

Supplement: S1 Fig — Average values of five experimental replicates are given for microcosms with (hemi)cellulose or starch, and of three experimental replicates from microcosms without added substrate. Error bars represent standard deviation. (TIF) [file pone.0212937.s002.tif]
