# Supplementary material for: Divergent microbial communities in groundwater and overlying soils exhibit functional redundancy for plant-polysaccharide degradation
Source: PLoS One. 2019 Mar 13;14(3):e0212937. doi: 10.1371/journal.pone.0212937 (PMC6415789; doi:10.1371/journal.pone.0212937)
Supplement: S1 Table — Given are average values and standard deviation. Gene copy numbers for enriched groundwater and soil samples have been determined by qPCR in triplicate reactions. Gene copy numbers for microcosms have been calculated based on the amount of enriched groundwater (250 L) or soil (10 g) used to in the microcosm setups. (PDF) [file pone.0212937.s003.pdf]

	bacterial 16S rRNA gene copies			
groundwater	$1.03 * 10^{7}$	±	$3.09 * 10^{5}$	L^{-1}
soil	$1.24 * 10^{13}$	±	$4.99 * 10^{12}$	kg^{-1}
groundwater microcosm	$2.598 * 10^{9}$	±	$7.73 * 10^{7}$	$microcosm^{-1}$
soil microcosm	$1.24 * 10^{11}$	±	$4.99 * 10^{10}$	$microcosm^{-1}$
