# Supplementary material for: Divergent microbial communities in groundwater and overlying soils exhibit functional redundancy for plant-polysaccharide degradation
Source: PLoS One. 2019 Mar 13;14(3):e0212937. doi: 10.1371/journal.pone.0212937 (PMC6415789; doi:10.1371/journal.pone.0212937)
Supplement: S2 Table — Taxonomic classification of the OTUs based on SILVA reference database release SSU 119 is shown on phylum and class level, as well as on the highest level up to genus where classification was possible. Levels of significance are *: p < 0.05, **: p < 0.01, and ***: p < 0.001, based on Breslow-Day test with Holm-Bonferroni correction. (PDF) [file pone.0212937.s004.pdf]

OTU	Taxonomy			Groundwater microcosms		Soil microcosms	
	Phylum	Class	Highest level	(hemi) cellulose	starch	(hemi) cellulose	starch
Otu00204	Acidobacteria	-	Paludibaculum		***		
Otu00109	Acidobacteria	-	Subgroup 2			***	***
Otu00130	Acidobacteria	-	Subgroup 2				***
Otu00172	Acidobacteria	-	Subgroup 2				***
Otu00239	Actinobacteria	-	Mycobacterium			**	
Otu00112	Actinobacteria	-	Cellulomonas		**		
Otu00018	Actinobacteria	-	Microbacterium		***		
Otu00007	Actinobacteria	-	Propionicicella	**	***		
Otu00088	Actinobacteria	-	Propionicicella		***		
Otu00001	Bacteroidetes	Bacteroidia	Paludibacter	***			
Otu00057	Bacteroidetes	Cytophagia	Emticicia		***		
Otu00024	Bacteroidetes	Cytophagia	Leadbetterella		***		
Otu00056	Bacteroidetes	Flavobacteriia	Flavobacterium		*		
Otu00327	Bacteroidetes	Flavobacteriia	Flavobacterium		*		
Otu00011	Bacteroidetes	Flavobacteriia	Flavobacterium	*			
Otu00019	Bacteroidetes	Sphingobacteriia	Sediminibacterium			***	
Otu00220	Bacteroidetes	Sphingobacteriia	Chitinophagaceae				*
Otu00066	Bacteroidetes	Sphingobacteriia	env.OPS 17				***
Otu00229	Bacteroidetes	Sphingobacteriia	NS11-12 marine group		**		
Otu00026	Bacteroidetes	Sphingobacteriia	Mucilaginibacter				***
Otu00068	Bacteroidetes	Sphingobacteriia	Mucilaginibacter				***
Otu00063	Bacteroidetes	Sphingobacteriia	Mucilaginibacter			***	
Otu00031	Bacteroidetes	Sphingobacteriia	Mucilaginibacter			*	
Otu00110	Bacteroidetes	Sphingobacteriia	Pedobacter	*			
Otu00864	Bacteroidetes	Sphingobacteriia	Sphingobacteriales		*		
Otu00107	Bacteroidetes	Sphingobacteriia	Sphingobacteriales	*			
Otu00044	Bacteroidetes	Sphingobacteriia	Sphingobacteriales			***	***
Otu00203	Bacteroidetes	Sphingobacteriia	Sphingobacteriales			***	
Otu00386	Bacteroidetes	-	WCHB1-32		***		
Otu00081	Chlamydiae	-	cvE6		*		
Otu00228	Chlamydiae	-	Simkaniaceae				***
Otu00016	Firmicutes	Clostridia	Clostridiaceae			***	***
Otu00023	Firmicutes	Clostridia	Clostridiaceae			***	***
Otu00075	Firmicutes	Clostridia	Clostridiaceae				***
Otu00086	Firmicutes	Clostridia	Clostridiaceae				***
Otu00197	Firmicutes	Clostridia	Clostridiaceae				**
Otu00010	Firmicutes	Clostridia	Clostridiaceae				***
Otu00159	Firmicutes	Clostridia	Clostridiaceae				***
Otu00097	Firmicutes	Clostridia	Clostridium				***
Otu00076	Firmicutes	Clostridia	Clostridium			***	
Otu00002	Firmicutes	Clostridia	Clostridium			***	***
Otu00036	Firmicutes	Clostridia	Fonticella			***	
Otu00045	Firmicutes	Clostridia	Fonticella			***	
Otu00083	Firmicutes	Clostridia	Clostridiales vadinBB60 grou[illegible]			***	
Otu00236	Firmicutes	Clostridia	Ruminiclostridium			***	
Otu00366	Firmicutes	Clostridia	Ruminiclostridium			*	
Otu00186	Firmicutes	Clostridia	Ruminiclostridium			***	
Otu00282	Firmicutes	Clostridia	Ruminococcaceae			***	
Otu00275	Firmicutes	Clostridia	Ruminococcaceae			*	
Otu00426	Firmicutes	Clostridia	Ruminococcaceae			*	
Otu00072	Firmicutes	Clostridia	ODP1230B8.23			***	*
Otu00073	Firmicutes	Clostridia	ODP1230B8.23			***	***
Otu00125	Firmicutes	Negativicutes	Veillonellaceae	*			
Otu00136	Firmicutes	Clostridia	Clostridia			*	*
Otu00027	Parcubacteria	-	Parcubacteria	***			
Otu00139	Planctomycetes	Planctomycetacia	Isosphaera			***	
Otu00141	Planctomycetes	Planctomycetacia	Planctomyces			***	
Otu00042	Proteobacteria	Alphaproteobacteria	Caulobacter		***		
Otu00060	Proteobacteria	Alphaproteobacteria	Bradyrhizobiaceae				***
Otu00334	Proteobacteria	Alphaproteobacteria	Ancalomicrobium		***		
Otu00051	Proteobacteria	Alphaproteobacteria	Pleomorphomonas		***		
Otu00251	Proteobacteria	Alphaproteobacteria	Roseiarcus			***	
Otu00062	Proteobacteria	Alphaproteobacteria	Variibacter			***	*
Otu00093	Proteobacteria	Alphaproteobacteria	DA111			***	
Otu01177	Proteobacteria	Gammaproteobacteria	CHAB-XI-27		***		
Otu00013	Saccharibacteria	-	Saccharibacteria	***	***		
Otu00005	Saccharibacteria	-	Saccharibacteria		**		
Otu00205	Saccharibacteria	-	Saccharibacteria	***			
Otu00318	Verrucomicrobia	Opitutae	Opitutus		***		
Otu00038	Verrucomicrobia	Spartobacteria	Terrimicrobium		***		
Otu00176	Verrucomicrobia	Spartobacteria	DA101 soil group			*	
